# Cup accuracy and early-term clinical outcomes of a novel, pinless, robotic-assisted total hip arthroplasty system: A first-in-human pilot study

**DOI:** 10.1186/s42836-025-00299-x

**Published:** 2025-03-20

**Authors:** David Liu, Atul F. Kamath, Jason Cholewa, Luminita Stoenica, Mike B. Anderson, Haig Lennox

**Affiliations:** 1 The Gold Coast Centre for Bone and Joint Surgery, 14 Sixth Avenue, Palm Beach, Gold Coast, 4224 Australia; 2 Cleveland Clinic Orthopaedic and Rheumatologic Institute, 9500 Euclid Avenue, Cleveland, OH 44195 USA; 3 Zimmer Biomet, Warsaw, IN 46580 USA; 4 Zimmer Biomet, 14167 Berlin, Germany

**Keywords:** Arthroplasty, Direct anterior approach, Acetabular cup, Total hip replacement, Robotic

## Abstract

**Background:**

Malpositioning of the acetabular cup represents a challenge during total hip arthroplasty (THA). The purpose of this study was to evaluate the accuracy of acetabular cup placement and early postoperative clinical outcomes with a novel, pinless, fluoroscopic-guided, robotic-assisted application for direct anterior (DA) approach THA.

**Methods:**

This prospective, pre-market phase 2 study enrolled 19 patients undergoing THA for osteoarthritis. Standing anteriorposterior (AP) and lateral radiographs up to 1 year postoperatively were assessed for component fixation and complications. Martell Hip Analysis software was used to assess radiographic acetabular anteversion and inclination from postoperative standing AP pelvis images and the results were compared to target and final component values from the surgical logs. Patient reported outcome measures (PROMs) were collected preoperatively, four weeks, three months, and one year after operation.

**Results:**

Mean absolute difference for anteversion and inclination from respective targets on intraoperative fluoroscopic views was 1.4° ± 1.3° (*P* = 0.159) and 1.3° ± 1.1° (*P* = 0.378). The absolute difference between postoperative radiographs and intraoperative target values was 2.91 ± 2.40° (*P* = 0.019) for anteversion and 3.84 ± 2.57° (*P* = 0.007) for inclination. The difference in target and postoperative radiographic inclination and anteversion was within 5° in 77.8% of cases, and the cup was within the Lewinnek safe zone in 16 of 18. Oxford Hip Score (OHS) (44.3 ± 4.6 vs. 22.2 ± 11.3), score on Numeric Pain Rating Scale (NRPS) (0.5 ± 1.0 vs. 5.7 ± 2.6), and Hip Osteoarthritis Outcome Score-12 (HOOS-12) Overall Score (91.4 ± 11.2 vs. 42.8 ± 20.1) were significantly improved at one year. At one-year, 88.2% and 11.8% of patients were very satisfied or satisfied.

**Conclusion:**

This first-in-human study on THA utilizing a pinless, fluoroscopy-based robotic arm demonstrated high accuracy in terms of radiographic inclination and anteversion, excellent hip-specific functional outcomes and safety one year after operation.

Video Abstract

**Supplementary Information:**

The online version contains supplementary material available at 10.1186/s42836-025-00299-x.

## Introduction

The volume of THA procedures has increased by approximately 14% between 2009 and 2015 [1], and the rate of revision THA has risen by 28.5% to 34.7% [2]. As the indications for THA include younger patients, demand for longevity and functionality of the implants is on the rise. This change in patient demographics and recreational behaviors partially explains the rise in revision surgeries with nearly 11% of primary THA patients reporting dissatisfaction, residual pain and functional limitations [3].

Acetabular cup malpositioning has been suggested as a major modifiable factor associated with poor PROMs, component wear, reduced range of motion (ROM), instability, and revision [4, 5]. To minimize dislocation, Lewinnek proposed an acetabular cup safe zone of 30° to 50° inclination and 5° to 25° anteversion [6], whilst Callanan recommended a more restricted safe zone of 30° to 45° inclination [7]. Recently, cup orientation philosophy has shifted from a prescriptive singular target to an individualized functional implant positioning based on patient phenotype, anatomical variations, and spinopelvic considerations. To complicate the issue further, differences in operative orientation when implanting the acetabular cup against radiographic orientation pose challenges to accurate manual placement of the cup [8]. Though formulas have been proposed to help surgeons correct for the difference in orientation angle between operative and radiographic angles [8], variabilities in component orientation due to inconsistencies in patient position and pelvic movement during the operation still occur [9].

Recent meta-analyses demonstrated superior reproducibility of robotic-assisted THA (raTHA), reporting an odds ratio of greater than 9.0 for cup placement within the Lewinnek safe zone compared to conventional THA (cTHA) [10, 11]. Most commercially available robotic systems use preoperative computed tomography (CT) for planning and placement of navigation pins for intraoperative positioning. Changes in operative steps compared to cTHA affect intraoperative workflow and increase surgical time [11]. Surgical approach also affects accuracy, with 2.0 greater odds of cup malpositioning with direct anterior approach (DA) versus posterior approach in cTHA [8]. Thus, the need for improved component placement in the DA approach is evident. A recent study comparing fluoroscopy-assisted with CT-based haptic DA raTHA found no significant difference in acetabular accuracy [12], questioning the use of haptic robotic guidance in DA THA. Another study found that surgeon estimation of acetabular cup orientation using intraoperative fluoroscopic imaging was unreliable [13] and recommended additional methods to optimize component position even when using intraoperative fluoroscopy.

A pinless fluoroscopy-based robotic arm system has been introduced recently and a preliminary cadaver and clinical studies showed that, compared to CT-based systems, the fluoroscopy-based system exposed patients to less radiation [14], was more efficient [15], had shorter operating time [16] and achieved superior accuracy and reproducibility [17]. Currently, one retrospective study has compared fluoroscopy-based raTHA to cTHA in humans and reported greater rates of cup placement within the Lewinnek safe zone (81.6% vs. 59.0%), and lower variance in cup positioning with raTHA [18]. However, the mean differences between target and radiographic angles were not reported, limiting the ability to interpret the magnitude to which raTHA was more precise. Preliminary evidence suggests less complications, pain, length of stay, and opioid use up to six-weeks postoperatively [19, 20]. The purpose of this study was to investigate the accuracy of cup placement with a pinless fluoroscopy-based robotic system and to analyze patient outcomes through a one-year follow-up.

## Methods

This was a prospective, single-arm, pre-market pilot study of patients who received raTHA conducted by two surgeons at a single institution in 2021. Ethical Approval for the study was obtained with the St John of God Health Care Human Research Ethics Committee (Project ID: 1749).

Study inclusion criteria included:

1. Osteoarthritis of the hip,
2. Suitable for direct anterior THA using cementless acetabular and femoral components,
3. Aged 18 to 80 years,
4. Body Mass Index (BMI) less than 40 kg/m^2^.

Study exclusion criteria were:

1. THA for acute femoral neck fracture, developmental dysplasia of hip, significant pelvic or proximal femoral deformity, osteoarthritis with in-situ metalware requiring removal,
2. Revision THA,
3. Patients with long-segmental spinal fusions,
4. Patient unable to give informed consent,
5. Patient unable or unwilling to return for all follow-up time points and imaging.

**Fig. 1:**
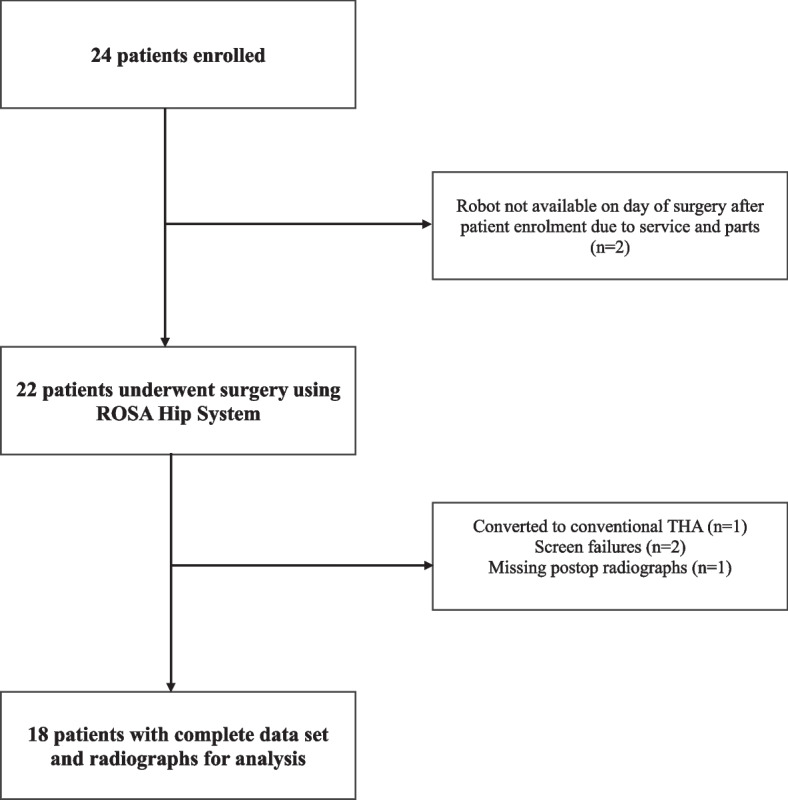
STROBE (Strengthening the Reporting of Observational Studies in Epidemiology) flow diagram of patient inclusion into the study analysis

Study enrollment is detailed in Fig. 1. The average age of the participants was 66.7 ± 7.9 years, average BMI was 28.1 ± 3.5 kg/m^2^, and they were predominantly males (78.9%).

All patients underwent DA raTHA with a pinless fluoroscopy-based robotic-arm orthopedic surgical assistance device (ROSA® Hip System v1.0, Zimmer Biomet, Warsaw, IN, USA) and were implanted with a G7® Acetabular and Taperloc® Femoral stem system (Zimmer Biomet, Warsaw, IN, USA). All cases included in the analysis were the first of a consecutive series with the ROSA Hip System. Thus, both surgeons were still within the 12-case learning curve for all cases reported in this study [16]. The intraoperative plan for inclination and version was recorded into the robotic system’s log files. Surgical time from incision to closure was recorded. The target zone for acetabular inclination was 30°–45° and for acetabular version was 5°–25° in anteversion for surgeon 1. Surgeon 2 used Formus Labs Hip Platform (Formus Labs Limited, Auckland, New Zealand) to plan a patient-specific cup alignment derived from the stem fit, hip center and combined version, resulting in a range of 16° to 25° for anteversion and 40° to 43° for inclination.

Standing anteroposterior (AP) radiographs were taken at four-weeks postoperatively and acetabular cup orientation was measured using the Martell Hip Analysis Suite™ (John Martell MD, Chicago, IL, USA). The Hip Analysis Suite™ requires the user to define the locations of the ischial tuberosities, acetabular cup, and femoral head to direct the automatic edge-detection functionality [14]. Inclination and version were read twice by two independent readers and results were averages of the two readings. Inter- and intra-rater reliability was assessed by having each reader re-evaluate the first 18 cases one week after the initial reading and the calculation of the concordance correlation coefficient (r_c_). The agreement level was classified as strong when r_c_ was 0.6 to ≤ 0.8; and as almost perfect when r_c_ > 0.8 [21]. With regard to intra-rater reliability, the r_c_ was 0.95 and 0.8 for reader 1 and 2 for anteversion, respectively and r_c_ was 0.97 and 0.78 for reader 1 and 2 for inclination, respectively. With inter-rater reliability r_c_ was 0.89 and 0.87 for anteversion and inclination, respectively.

The outliers of accurate acetabular cup placement were defined as an absolute difference in either inclination or anteversion of more than 5° from the planned angle and the overall success rate was calculated based on the percentage of participants whose cups were within Callanan [7] and Lewinnek [6] safe zone for inclination.

Clinical outcomes were assessed in terms of adverse events and survivorship, defined as the revision of any component. PROMs were collected preoperatively and at four-weeks, three-months, and one-year postoperatively, including OHS, HOOS-12, NPRS, and a 5-point Likert scale (patient satisfaction). Minimal clinical important changes (MCICs) for the OHS, HOOS-12, and NPRS had been reported to be 8 points [22], 24 points [23], and 2 points [24], respectively.

### Surgical technique

**Fig. 2:**
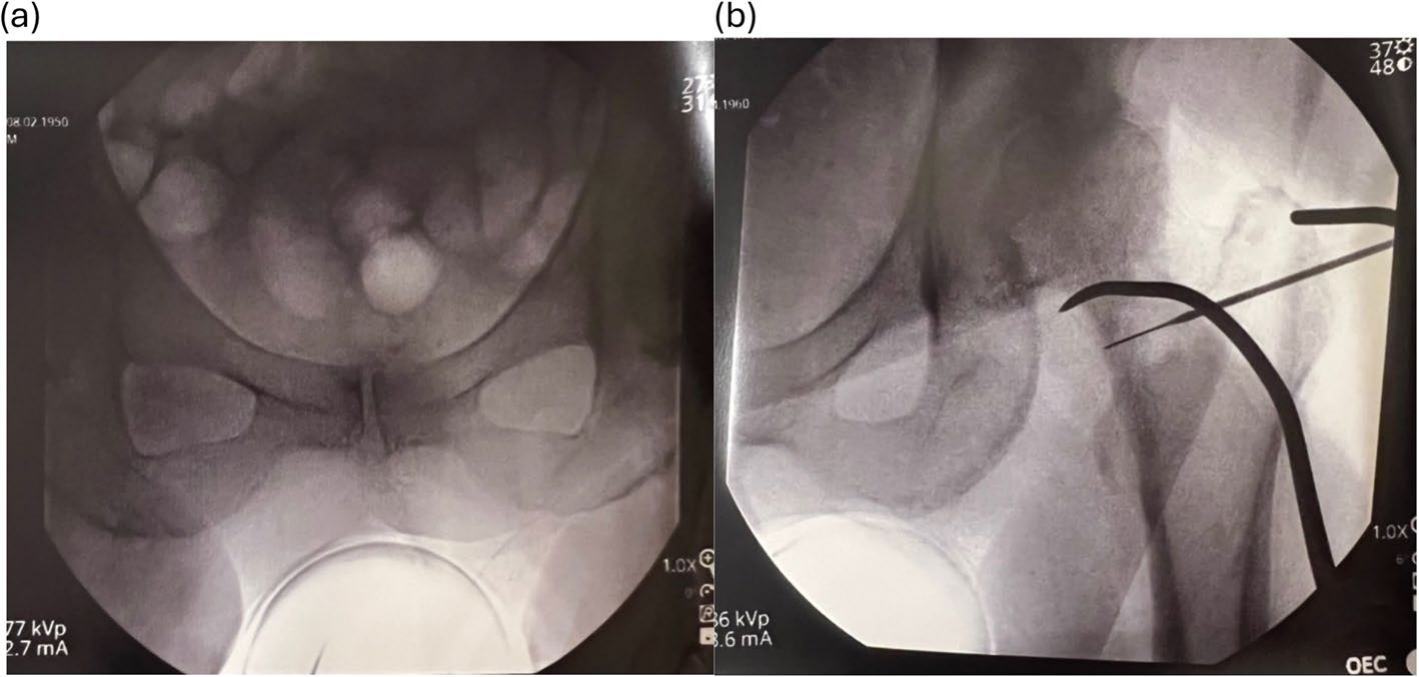
Intraoperative image intensifier radiographs of the leveled pelvis (**a**) and AP of hip (**b**) are obtained for surgical navigation with the ROSA hip system

**Fig. 3:**
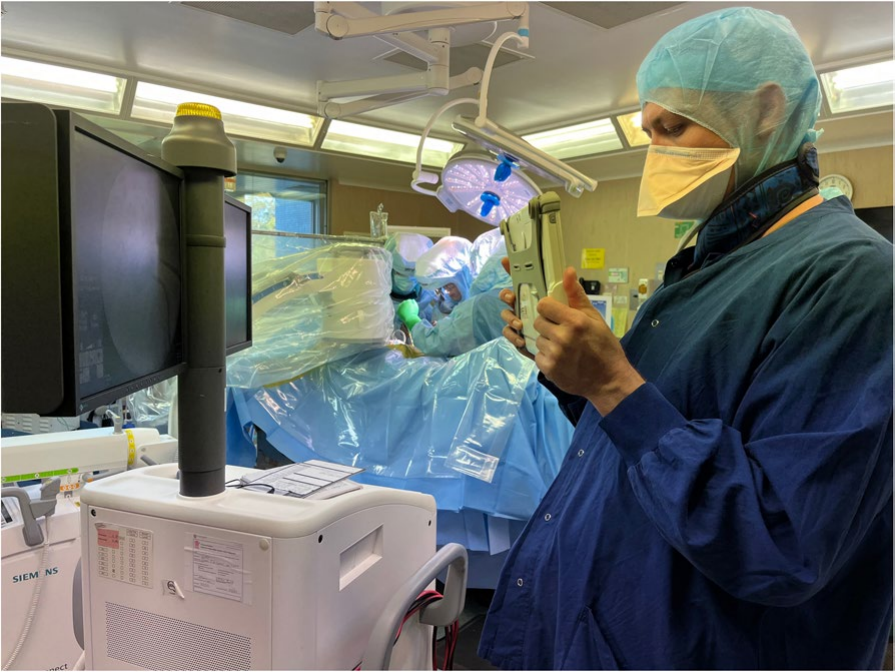
Images on the C-arm monitor are captured using the ROSA tablet by the ROSA support team and then transferred to the robotic system

**Fig. 4:**
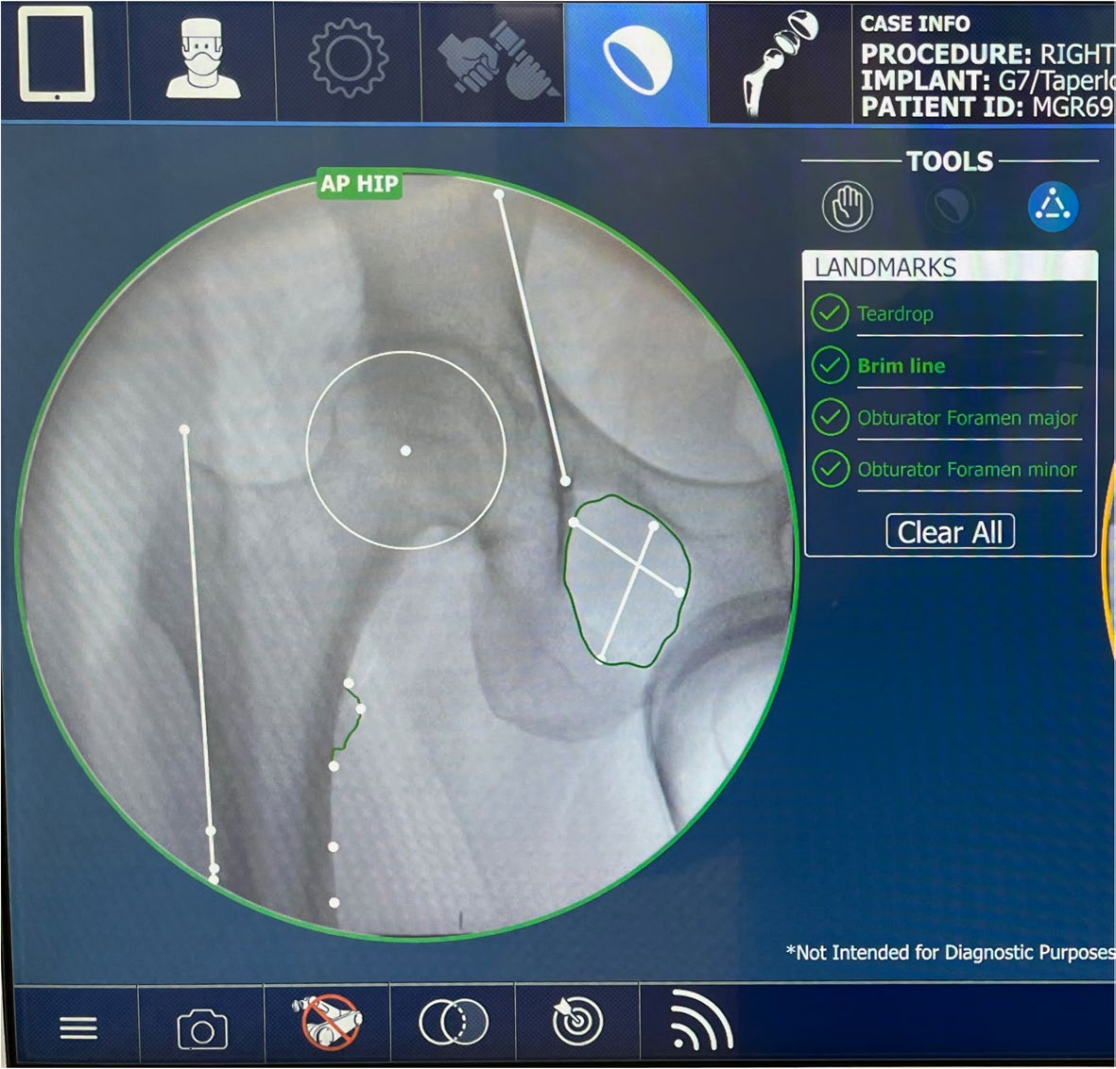
Anatomical landmarks on AP hip image required for surgical navigation and robotic arm guidance is automatically populated with surgeon verification

**Fig. 5:**
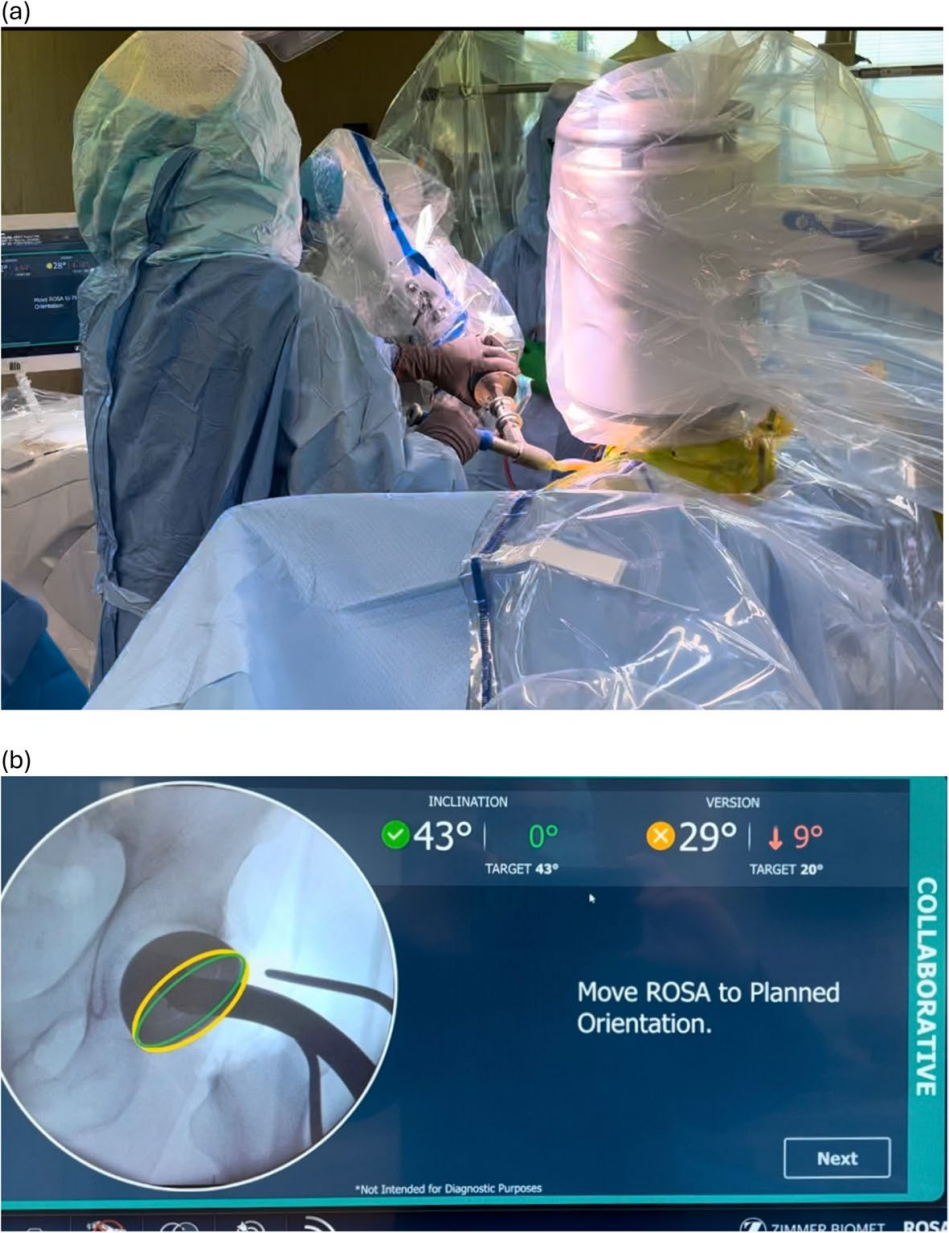
The ROSA robotic arm moves with surgeon collaboration (**a**) and haptic, visual and auditory feedback to the targeted inclination and version angles (**b**)

**Fig. 6:**
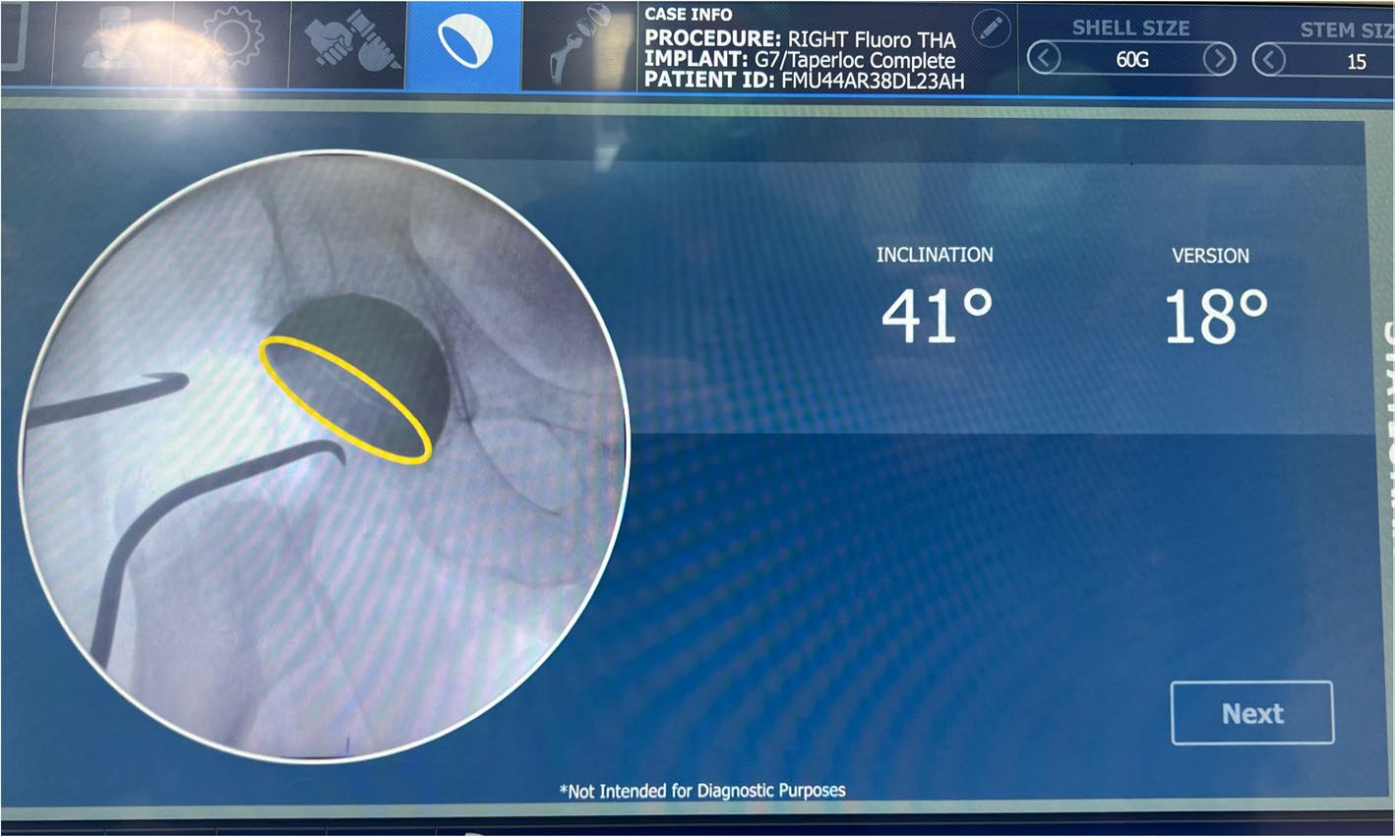
Final intraoperative cup position was validated and recorded into robotic surgical log files

**Fig. 7:**
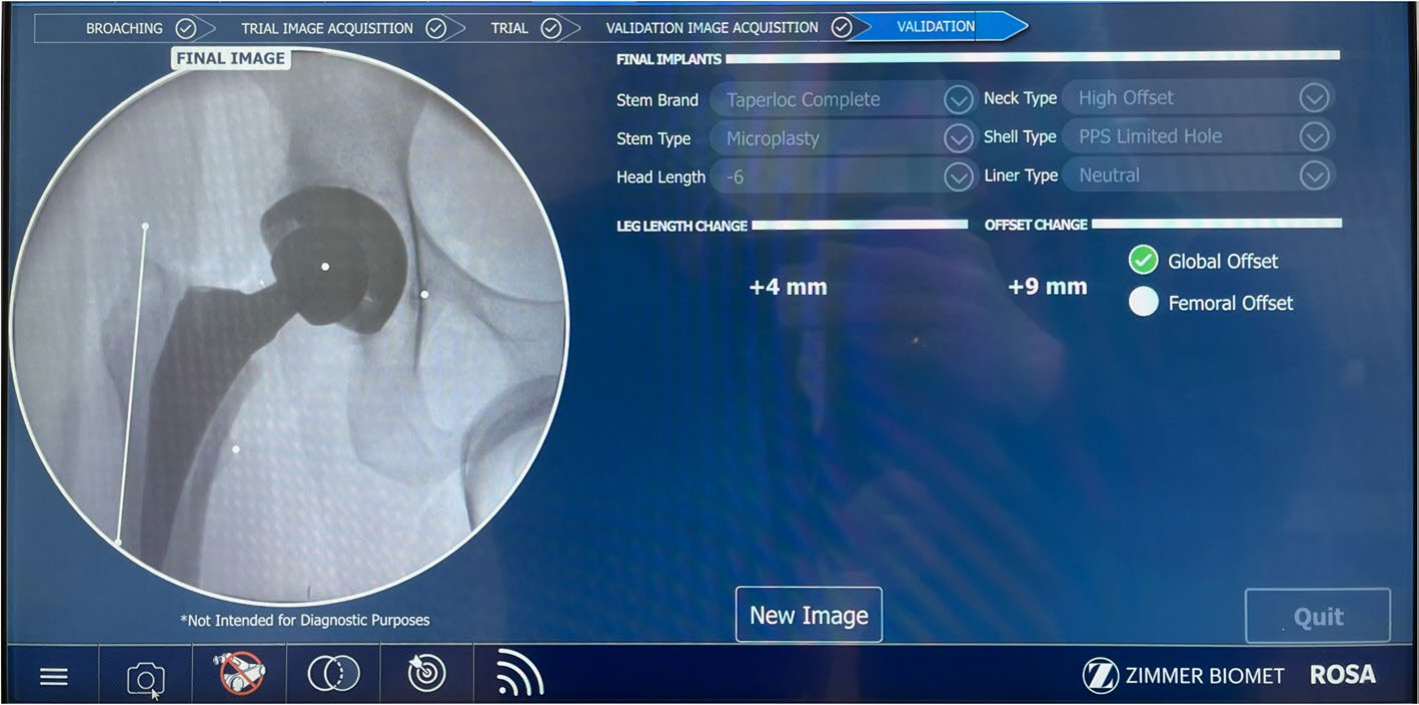
Following final femoral component implantation, measurements of change in leg length, femoral offset and global offset are recorded

The ROSA hip system was designed as a fluoroscopy-based pinless robotic system for DA THA. No trackers are attached to the pelvis or femur, nor is preoperative CT scanning required as the robot utilizes landmarking derived from intraoperative image intensifier (II) pictures (Fig. 2). In the supine position, a fluoroscopic reference image of the levelled pelvis was obtained, aiming for the same pelvic tilt as the preoperative standing AP pelvic radiograph. This was achieved by rotating the C-arm until the pelvic inlet and obturator foramina on the II image matched the preoperative AP standing radiograph. An AP hip reference image was then acquired and a photo of both images on the C-arm monitor was captured using the ROSA tablet (Fig. 3), and then transferred to the robotic system for automated landmarking with surgeon verification (Fig. 4). Femoral head resection and reaming were performed manually. The cup was then attached to the robotic arm, which was then moved, with surgeon collaboration, to the targeted inclination and version angles for seating (Fig. 5). Once the target cup alignment was reached, the robot arm was locked into the achieved position for rigid stability during impaction. After the cup was seated, the robotic arm was released from the cup impactor. The cup was checked for primary stability and a further II image was taken for final cup position validation (Fig. 6). Femoral broaching was also performed manually, and further fluoroscopic images were obtained during trialing, and following final femoral component implantation for implant validation and final measurement of change in leg length, femoral offset and global offset (Fig. 7). The robotic software uses a radiographic overlay technique of initial reference image to validation image to calibrate and calculate the measurements. The intraoperative plan and validated measurements for cup inclination and anteversion were recorded into the robotic system’s log files.

The ROSA hip system was developed specifically to provide minimal change to workflow for surgeons who perform DA THA using fluoroscopic assistance. Advantages include surgical efficiency given nominal alterations to normal technique, no need for trackers or pin insertion into target bones or intraoperative bone landmarking, minimal learning curve and requiring no preoperative CT scan. Disadvantages include restricted use to fluoroscopically-guided DA THA only, sensitivity to quality of II images, and manual femoral neck osteotomy, acetabular reaming and femoral broaching.

### Data analysis

A power calculation was performed based on previous studies using the published difference in acetabular cup angles between navigation and conventional instrumentation. Using a mean difference from planned acetabular inclination of 2.65° ± 1.85° in prior navigation studies compared to a mean difference of 4.625° ± 3.425° using conventional instrumentation [25, 26], with 80% power and alpha set at 0.05, the number of patients needed to detect significance between robotic and conventional instrumentation was nine. With similar parameters, a sample size of 11 patients was needed to show an improvement of 20 points (SD 9.5) in the OHS from preoperative to postoperative one year, taking a minimally important change of 8 points as clinically relevant [22]. Differences in planned angles versus robotic validated and postoperative radiographically-measured angles were assessed by paired samples t-tests. PROMs were assessed with the Sign-rank test. Continuous data were presented as means ± standard deviation (SD), and categorical data were reported as frequencies and percentages. The alpha was set at *P* < 0.05.

## Results

There were no significant differences (Table 1) between the final planned targeted angle and intraoperative robotic validated angle for anteversion (*P* = 0.159) or inclination (*P* = 0.378). Significant differences were found between the targeted angle and the postoperative radiographic angles (Table 2) for anteversion (*P* = 0.019) and inclination (*P* = 0.007). There were no significant (*P* = 0.241) differences between the intraoperative and postoperative radiographic angle for anteversion; however, the respective differences for inclination were significant (*P* < 0.001) (Table 3).

**Table 1:** Accuracy of reproducing the intraoperative plan as assessed by the final intraoperative validated angles^1^. Data are presented as means ± standard deviation (95% confidence intervals)

	**Final Planned Target Angle**	**Intraoperative Validated Angle**	***P*-value**	**Mean Absolute Error**
Anteversion	19.1° ± 2.6° (17.8° – 20.3°)	19.7° ± 3.0° (18.2° – 21.1°)	0.159	1.4° ± 1.3° (0.8° – 2.0°)
Inclination	40.9° ± 1.1° (40.4° – 41.4°)	40.6° ± 2.2° (39.5° – 41.6°)	0.378	1.3° ± 1.1° (0.8° – 1.8°)

*Validated using the robotic software*

**Table 2:** Accuracy of reproducing the targeted plan as assessed by the postoperative radiographs. Data are presented as means ± standard deviation (95% confidence intervals)

	**Final Planned Target Angle**	**Postoperative Radiographs**	***P*-value**	**Mean Absolute Error**
Anteversion	19.1° ± 2.6° (17.8° – 20.3°)	21.0° ± 3.9° (19.04° – 22.9°)	0.019	2.9° ± 2.4° (1.7° – 4.1°)
Inclination	40.9° ± 1.1° (40.4° – 41.4°)	43.56° ± 4.13° (41.5° – 45.6°)	0.007	3.8° ± 2.6° (2.6° – 5.1°)

**Table 3:** Accuracy of reproducing the intraoperative validated angle as assessed by the post-operative radiographs^1^. Data are presented as means ± standard deviation (95% confidence intervals)

	**Intra-operative Validated Angle**	**Post-operative Radiographs**	***P*-value**	**Mean Absolute Error**
Anteversion	19.7° ± 3.0° (18.2° – 21.1°)	21.0° ± 3.9° (19.04° – 22.9°)	0.241	3.1° ± 2.5° (1.9° – 4.4°)
Inclination	40.6° ± 2.2° (39.5° – 41.6°)	43.56° ± 4.13° (41.5° – 45.6°)	< 0.001	3.9° ± 2.2° (2.8° – 5.0°)

*Validated using the robotic software*

**Fig. 8:**
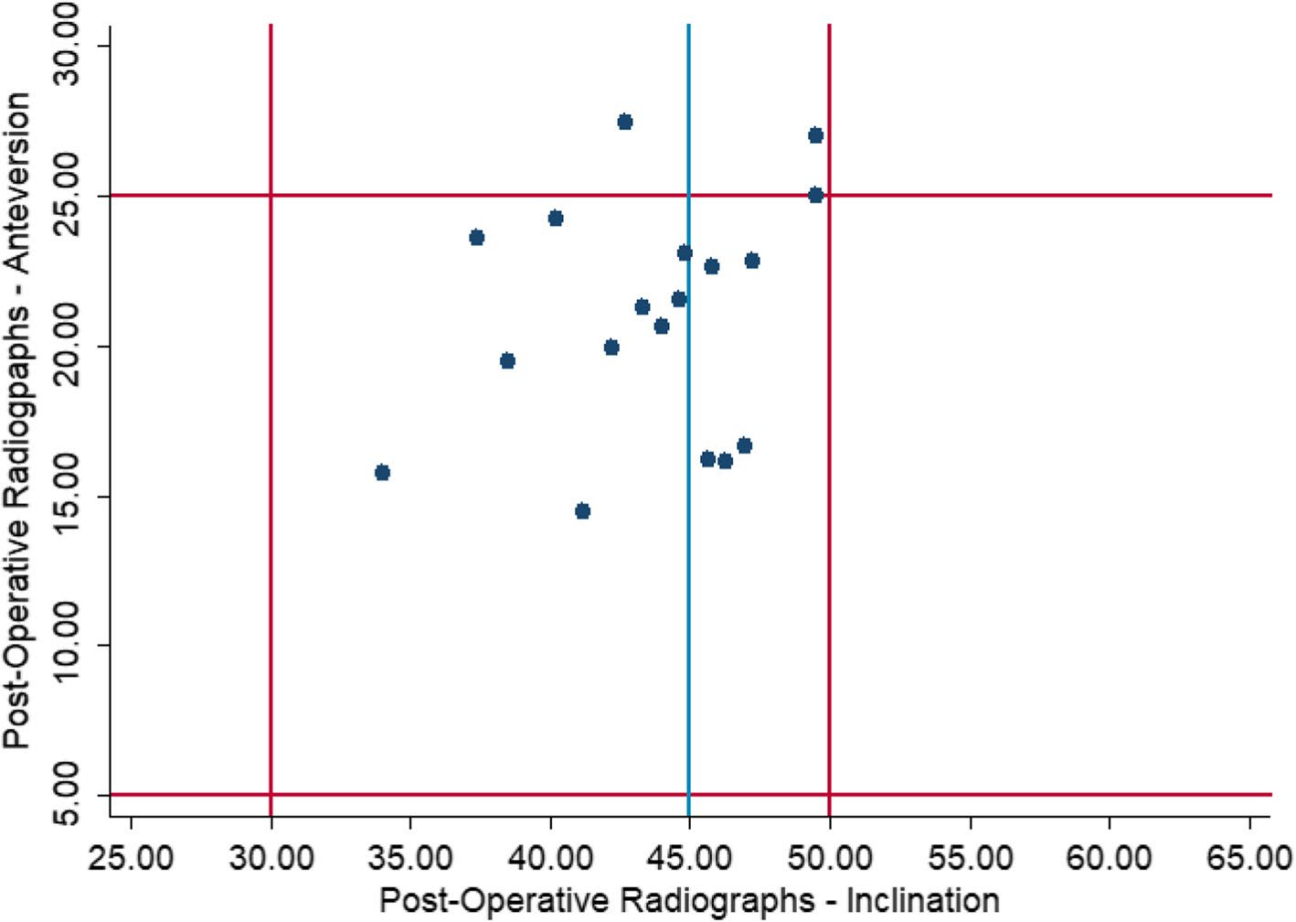
Scatter plot demonstrating the implants placed within the Lewinnek safe zones (red lines) according to postoperative radiographic analysis. The blue line indicates the upper limit of the Callanan safe zone for acetabular inclination. Values in degrees

On postoperative radiographs, 14 of 18 (77.8%) acetabular cups were placed within 5° of the targeted anteversion and inclination angles. In 16 of 18 cases (88.9%), the cups were within the Lewinnek safe zone (Fig. 8). In 11 of 18 cases (61.1%), the cups were within the Callanan safe zone (88.9% within the anteversion safe zone and 61.1% within the inclination safe zone).

There was a significant (*P* < 0.001) improvement between preoperative and 4-week, 3-month, and 12-month outcomes in terms of scores of OHS, all HOOS-12 subscales, and NRPS (Table 4). Mean surgical time was 95.2 ± 21.9 min. There was significant (*P* < 0.0001) difference in operating time between surgeon one (*n* = 9, 76.4 ± 6.2 min) and surgeon two (*n* = 12, 112.6 ± 15.0 min). Flexion contractures were present in 3 of 19 patients preoperatively and resolved in all patients during the follow-up. Leg length discrepancies were found in 9 of 19 patients preoperatively and were corrected in all patients during the follow-up. There were no cases of fracture, acetabular migration, femoral subsidence, bone reabsorption or radiolucency greater than 2 mm at any follow-up time point. One case had heterotopic ossification at one-year follow-up, and one patient reported ongoing pain and hip stiffness at four-weeks postoperatively that resolved by one-year after operation. There were no revisions during the follow-up.

**Table 4:** Patient reported outcome measures

	**Preoperative**	**Postoperative 4-weeks**	**Postoperative 3-months**	**Postoperative 12-months**
Patient Satisfaction	NA	Satisfied: 6/19 (31.6%) Very Satisfied: 13/29 (68.4%)	Very Dissatisfied: 1/18 5.6%) Neutral 1/18 (5.6%) Satisfied 3/18 (16.7%) Very Satisfied 13/18 (72.2%)	Satisfied: 2/17 (11.8%) Very Satisfied 15/17 (88.2%)
OHS	22.2 ± 11.3	37.4 ± 5.5	39.7 ± 7.3	44.3 ± 4.6
NPRS	5.7 ± 2.6	1.4 ± 1.5	1.2 ± 1.4	0.5 ± 1.0
HOOS-12				
Overall	42.8 ± 20.1	74.6 ± 14.3	82.6 ± 16.0	91.4 ± 11.2
QoL^1^	35.7 ± 19.1	68.8 ± 19.0	77.8 ± 23.1	89.7 ± 15.6
Function	47.0 ± 23.4	79.9 ± 13.1	87.2 ± 13.3	91.5 ± 11.0
Pain	41.0 ± 21.7	75.0 ± 16.1	83.0 ± 15.6	93.0 ± 10.6

## Discussion

The main finding of this study was that DA THA with a novel pinless fluoroscopy-based robotic assistance is accurate and reliable, allowing for control of planned cup position through collaborative feedback of real-time position and tight control when impacting the cup. There was no significant difference between the intraoperatively planned and achieved cup inclination and anteversion, as validated using intraoperative fluoroscopy. When comparing the targeted angles to the radiographic results, we found that in 88.9% of cases, the cup was within the Lewinnek safe zone, 61.1% of cases within the Callanan safe zone, and 77.8% of cases were within 5° of the targeted angles for inclination and anteversion, indicative of high accuracy. There were no revisions or dislocations through the first postoperative year.

This study has some limitations. First, we did not include a control group of non-robotic cases. However, the current study reported the results of a phase 2 clinical trial, which aimed to evaluate effectiveness and side effects in a small number of patients. There was a complete data set for 18 patients, with no patients lost to follow-up and all radiographic measurements were independently undertaken by 2 separate surgeons on 2 separate occasions. The focus of the current study was not to compare robotic with non-robotic system but rather measure the efficacy of the ROSA hip system by comparing the difference between intraoperative robotic parameters and achieved cup alignment. As the first in-human pilot study, the study enrolled a small number of patients, to ensure safety and efficacy of the robotic system, in keeping with the methodology of a phase 2 trial where one responsibility of the clinical trial team is to limit the number of patients who might potentially be subjected to harmful exposure of an unproven technology in the clinical setting. To accomplish safe graduated introduction of new technology, the ROSA hip system now needs to undergo a phase 3 trial in larger samples to confirm its efficacy compared to standard instrumentation.

Additionally, the effects of pelvic tilt and X-ray offset on scan-rescan reproducibility of hip radiographic imaging must be considered before interpreting the results. Each 1° change in pelvic tilt leads to a 0.5° to 1° change in anteversion [27] and an approximately 0.5° change in inclination [28]. Similarly, X-ray offset is also known to affect the inclination angle measured radiographically [29]. Several corrective methods have been developed to minimize the calculation bias but showed high degrees of between-measurement variability and inconsistent agreement with gold standard CT scans [29]. Mean differences between correction methods for measuring anteversion on AP radiographs have been reported to range from 0.9° to 5° [30]. It is also well established that pelvic tilt changes between standing, sitting and supine positions [31]. The robotic assistance in the present study calibrated intraoperative supine fluoroscopic images against a preoperative standing AP radiograph. While standing AP radiographs were used to measure angles postoperatively, we were unable to control for differences in X-ray offset and pelvic tilt between pre- and postoperative X-rays, which may explain the greater difference between target angles and postoperative inclination and anteversion compared to intraoperative measurements. Moreover, THA itself may affect pelvic tilt. Blondel et al. reported a mean change in standing pelvic tilt of 3.0° ± 0.3° following THA [32], and other authors reported that THA significantly affected pelvic tilt, with the greatest changes occurring within the first postoperative year [33].

Another limitation is that both participating surgeons were subspecialty fellowship-trained orthopedic surgeons with 20 years’ experience focusing on hip and knee arthroplasty. Each surgeon performs between 100 and 120 primary THA per year, with most DA THA performed under fluoroscopic guidance. One surgeon had over 5-year experience with DA THA whilst the other had been performing DA for 1 year before study commencement. The study was conducted at a high-volume private hospital that performs about 500 THAs per year. The experience and volume of the participating surgeons and hospital may have biased the results, and similar results may not be reproducible by other surgeons or in other hospitals. Neither surgeon had any clinical experience with ROSA hip nor hip robotics prior to the study.

The mean difference between planned and radiographic implantation angles in this study was 3.8° and 2.9° for inclination and version, respectively. Whilst the differences in targeted and postoperative radiographic anteversion and inclination were statistically significant, we do not believe mean difference of 1.0 degrees anteversion and 2.66 degrees inclination was clinically meaningful. The reason for the difference, we believe, is a combined result of robotic precision and the difference in pelvic tilt between the intraoperative and postoperative anteroposterior (AP) pelvic radiographs. Three other studies have reported similar accuracy of this fluoroscopic-based raTHA system. Ong et al. [34] reported mean differences of 2.25° and 4.09° for inclination and anteversion in non-obese patients. Buchan et al. [35] reported significantly more raTHA cases were within the Lewinnek safe zone compared to cTHA (81.6% vs. 59.0%) and a significantly smaller variance for both inclination and anteversion with raTHA. In cadavers, Kamath et al. [17] reported a mean difference between planned and implantation angle for inclination and version of 1.8° ± 1.3° and 2.6° ± 2.3° and 100% of raTHA cases within the Callanan and Lewinnek safe zones. Methodological differences in the measurement of the final implantation angles may partially explain the differences in accuracy between human and cadaver studies. The present study relied upon postoperative radiographs and a computer-assisted algorithm to correct for pelvic tilt and X-ray offset. Although this method is more accurate than conventional radiographic measurement of inclination and version [36], Kamath et al. [17] used a calibrated coordinate measurement machine and radio-opaque markers to obtain intraoperative images at different steps in the surgical process, thereby reducing measurement bias due to differences in pelvic tilt.

Freehand placement of the acetabular cup via the DA approach is associated with a greater risk of cup malposition compared to a posterior approach [7], though the use of intraoperative fluoroscopic imaging restores positioning accuracy with the DA approach [37]. Although meta-analysis results reported superior accuracy with raTHA, there is evidence to suggest more variability and lower accuracy with robotic-assisted DA compared to robotic-assisted posterior approach for both anteversion and inclination [38, 39]. Our results were comparable to previous raTHA studies that employed DA THA using a CT-based raTHA system [12, 38, 39]. Domb et al. [39], Redmond et al. [38] and other authors have reported similar results with 72% of cases being within the Callanan safe zone and mean differences between target and radiographic for anteversion and inclination being 3.64° and 3.80°, respectively [12]. It should be noted that, in the present study, the targeted inclination with surgeon 2 was at the upper threshold of the Callanan safe zone (40°–43°), which may explain the lower percentage of cases being within the safe zone for inclination compared to anteversion. The results of the current study against previous raTHA studies are summarized in Table 5.

**Table 5:** Summary of achieved cup accuracy from previous published studies using ROSA or MAKO raTHA compared to the current study

Author	Robotic System	Mean error between target and achieved alignment		Percentage within safe zone	
		**Inclination**	**Anteversion**	**Lewinnek**	**Callanan**
Liu (Current Study)	ROSA	3.8° ± 2.6°	2.9° ± 2.4°	88.9%	61.1%
Kamath [17]	ROSA Cadaveric	1.8° ± 1.3°	2.6° ± 2.3°	NR	100%
Buchan [35]	ROSA	2.8° ± 5.2°	3.5° ± 5.1°	81.6%	NR
Ong [34]	ROSA	2.25° ± 5.55°	4.09° ± 6.14°	77%	NR
Redmond [38]	MAKO	3.3° ± 3.1°	2.9° ± 2.3°	NR	NR
Domb [39]	MAKO	Posterior	Posterior	Posterior	Posterior
		0.13° ± 3.33°	4.89° ± 3.87°	97.78%	94.07%
		Anterior	Anterior	Anterior	Anterior
		0.78° ± 4.88°	0.56° ± 4.81°	87.10%	77.42%
Stewart [12]	MAKO	3.80° ± 4.41°	3.64° ± 3.13°	75%	NR

At 12 months postoperative, 100% of patients in the present study were either satisfied or very satisfied. The change in OHS, NPRS, and HOOS-12 scores from preoperative 22, − 5.2, and 48.9 all surpassed the MCIDs of 8 points [22], 2 points [24], and 24 points [23] for their respective measures. To our knowledge, this was the first study to evaluate PROMs over a perios of one-year with a fluoroscopic-based raTHA system. Studies with CT-based raTHA systems have reported similar findings, including one-year postoperative OHS scores of 40.8 [40] and HOOS-JR score of 86.0 [41]. Some [40, 41], but not all [42, 43], have reported superior PROMs with raTHA compared to non-raTHA results.

The mean operating time in the present study was 96.3 min, which was considerably longer than both the learning phase (44.3 ± 4.4 min), proficiency phase (38.0 ± 7.1 min), and longest operative time (69 min) reported by Buchan et al. for this system [16]. The operative time not only reflects surgeon experience, but also radiographer proficiency and ROSA support staff’s skill with image transfer and landmarking. None of the team involved with the study had any in-person hands-on training with the system prior to the first use and therefore the operative time very much reflect the learning curve with the system for all involved surgical team members. Buchan et al. [16] reported a learning curve of 12 cases and showed that, despite both surgeons still being within the learning curve, accurate cup placements were still achieved. There were also significant differences in the mean operative time between the two surgeons, being approximately 35 min. The longer operative time with surgeon 2 was ascribed to difficulty in the transfer of images from the fluoroscopic machine to the raTHA hip system on 2 occasions. Of note, surgeon 2 was within the operative learning curve of 100 cases [43] for the DA approach, which might contribute to the longer operative time irrespective of robotic adoption. Finally, both surgeons only received virtual training for the robotic platform, as COVID-19 pandemic restrictions prohibited in-person training. Regardless, it was determined that this first in-human pilot study was necessary to evaluate this pinless fluoroscopic-based robotic system. Since this study, version 1.1 of the system received FDA clearance and includes additional visual landmarking guides to aid the surgeon.

## Conclusion

This study demonstrated high accuracy in terms of radiographic inclination and version with a pinless, fluoroscopic-guided robotic application for DA THA. The results showed excellent hip-specific functional, safety and efficacy outcomes associated with raTHA over a one-year follow-up.

## Data Availability

The data and study materials are available on request.
